# Long-Term Health Consequences of SARS-CoV-2: Reaction Time and Brain Fog

**DOI:** 10.3390/neurolint18010006

**Published:** 2025-12-26

**Authors:** Ana Lesac Brizić, Branislava Popović, Tina Zavidić, Nevena Todorović, Verica Petrović, Nataša Pilipović-Broćeta, Ana R. Miljković, Aleksandar Ljubotina, Ema Dejhalla

**Affiliations:** 1 Faculty of Medicine, University of Rijeka, 51000 Rijeka, Croatia; ana.lesac@outlook.com (A.L.B.); branislava.popovic@medri.uniri.hr (B.P.); tina.zavidic@medri.uniri.hr (T.Z.); alexandar_ljubotina@yahoo.com (A.L.); 2 Health Center of Primorje-Gorski Kotar County, 51000 Rijeka, Croatia; 3 Family Medicine Office Branislava Popović, 51000 Rijeka, Croatia; 4 Istrian Health Centers, 52426 Lupoglav, Croatia; 5 Family Medicine Department, Faculty of Medicine, University of Banja Luka, 78000 Banja Luka, Bosnia and Herzegovina; nevena.todorovic@med.unibl.org (N.T.); verica.petrovic@med.unibl.org (V.P.); natasa.pilipovic-broceta@med.unibl.org (N.P.-B.); 6 Family Medicine Teaching Centre, Primary Health Care Centre Banja Luka, 78000 Banja Luka, Bosnia and Herzegovina; 7 The Medical Faculty in Novi Sad, University of Novi Sad, 21000 Novi Sad, Serbia; ana.miljkovic@mf.uns.ac.rs; 8 Community Health Centre Novi Sad, 21000 Novi Sad, Serbia; 9 Family Medicine Office Aleksandar Ljubotina, 51000 Rijeka, Croatia; 10 Medical Centre for Occupational Health Rijeka, 51000 Rijeka, Croatia

**Keywords:** cognitive dysfunction, COVID-19, mental fatigue, neurologic manifestations, reaction time

## Abstract

**Background/Objectives**: Beyond respiratory problems, COVID-19 can cause a variety of symptoms, such as neurological disorders caused by biological and psychological factors. Brain fog (BF), a post-illness cognitive impairment that many patients report, can be evaluated with reaction time (RT) testing. Response latency is measured by RT, which can be either simple (sRT) or complex (cRT). This study focuses on how COVID-19 affects cognitive function, with particular attention on RT changes, BF prevalence, and implications for daily life. **Methods**: The study included 599 participants from Bosnia and Herzegovina, Croatia and Serbia. RT was measured using PsyToolkit and participants completed a COVID-19-associated BF questionnaire. Participants who experienced BF after their latest COVID-19 infection rated its severity using a visual analogue scale (VAS). Additional clinical data were obtained from medical records. **Results**: BF was reported by 40% of participants post-COVID-19. Men reported it less frequently but found it more disruptive. RT progressively declined post-infection, reaching peak impairment at 15 weeks, following recovery, with RT normalizing by six months. **Conclusions**: COVID-19 is linked to temporary RT impairment, peaking at 15 weeks post-infection and resolving by six months, independent of BF presence. This study emphasizes the need for a biopsychosocial approach to BF management. Easily available RT assessments should be incorporated into routine clinical practice.

## 1. Introduction

The COVID-19 pandemic presents a range of complex biopsychosocial challenges, affecting individuals in various ways. Its primary clinical manifestation is a respiratory infection, as the SARS-CoV-2 virus binds to the angiotensin-converting enzyme 2 (ACE2) receptor, which is predominantly expressed on endothelial cells of the respiratory system and type 2 alveolar cells [1]. This mechanism explains the predominance of respiratory symptoms in COVID-19 [2,3,4,5,6]. However, due to the widespread distribution of ACE2 receptors, SARS-CoV-2 also affects multiple organ systems, leading to extrapulmonary complications, including neurological impairments. Among these, brain fog (BF) has emerged as a significant concern, characterized by confusion, fatigue, and cognitive dysfunction, affecting memory, concentration, and mental clarity. Notably, BF can persist even in individuals who experience mild or asymptomatic cases of COVID-19 [7].

In 2021, the National Institutes of Health (NIH) introduced the term “post-acute sequelae of SARS-CoV-2” (PASC) to describe the prolonged effects of the virus. PASC encompasses a spectrum of persistent symptoms, including respiratory difficulties, mobility impairments, mental health disturbances, fatigue, and cognitive dysfunction such as BF [8,9]. The link between COVID-19 and BF has become a prominent research focus, with reports suggesting that even after recovering from the acute phase of illness, some individuals continue to experience BF symptoms and other cognitive impairments [10].

Reaction time (RT) is a crucial measure of cognitive function, assessing the latency of response to stimuli. RT evaluation involves assessing attentional processes, memory, and decision-making capabilities. Simple reaction time (sRT) measures the response to basic stimuli, while complex reaction time (cRT) requires higher cognitive processing, including decision-making. COVID-19-related changes in RT may serve as an indicator of neurological or cognitive dysfunction, providing insights into attentional capacity, memory retention, and executive function [11].

Growing evidence suggests that SARS-CoV-2 infection is associated with persistent cognitive symptoms, including impaired attention, mental fatigue, reduced processing speed, and executive dysfunction, even in non-hospitalized individuals [12]. Several studies have reported prolonged cognitive deficits months after acute infection, often described by patients as brain fog. Reaction time (RT) testing has been widely used as an objective indicator of cognitive processing speed and attentional efficiency in neurological and post-infectious conditions. In post-COVID-19 research, prolonged RT has been associated with subjective cognitive complaints, fatigue, and reduced daily functioning. Therefore, RT assessment represents a practical and accessible tool for quantifying subtle cognitive impairments related to brain fog, particularly in primary care settings [13].

The aims of this study were to evaluate whether COVID-19 has short-term, post-acute, or long-term effects on cognitive function by examining RT and the presence of cognitive dysfunction, to determine the prevalence of BF among individuals recovering from COVID-19, and to investigate the impact of BF on daily life.

## 2. Materials and Methods

### 2.1. Participants

This cross-sectional mixed-methods study was conducted between May and September 2023 in eight family medicine offices located in three cities: Rijeka (Croatia), Banja Luka (Bosnia and Herzegovina), and Novi Sad (Serbia). Participants were recruited from patient lists of participating family medicine practices using a random number selection method.

Eligible participants were adults aged 18–60 years who voluntarily agreed to participate in the study. Individuals were excluded if they had a history of cognitive impairment, hospitalization due to COVID-19, or medical conditions known to affect cognitive function, motor coordination, or reaction time. Exclusion criteria included neurological disorders (e.g., multiple sclerosis, Parkinson’s disease, stroke, Alzheimer’s disease), acute headache disorders, psychiatric illnesses, and the use of medications that could impair motor speed or coordination.

Initially, 613 participants were recruited. Fourteen participants (eight from the COVID-19 group and six from the control group) chose not to complete the study. The final sample consisted of 599 participants, including 299 individuals with a history of COVID-19 infection and 300 control participants without prior COVID-19 infection. Each participant completed the assessment in approximately 10 min.

### 2.2. Reaction Time (RT)

Reaction time (RT) was assessed using PsyToolkit, a validated web-based platform for conducting cognitive and psychological experiments. Both simple reaction time (sRT) and complex reaction time (cRT) tasks were administered.

For the simple reaction time task, participants were instructed to press the space bar on a computer keyboard as quickly as possible each time a visual stimulus appeared on the screen. This task assessed basic sensorimotor response speed.

For the complex reaction time task, participants were presented with one of ten randomly displayed letters on the screen and were required to press the corresponding key on the keyboard. This task required higher cognitive processing, including stimulus discrimination, decision-making, and response selection.

Reaction time data were automatically recorded by the PsyToolkit software (version 0.6). The total duration of the RT assessment was approximately five minutes per participant.

### 2.3. Cognitive Impairment Questionnaire

Subjective cognitive impairment related to COVID-19 was assessed using an in-house questionnaire developed to capture commonly reported symptoms of post-COVID brain fog described in the literature. The questionnaire assessed the number of COVID-19 infections, perceived severity of acute illness, time elapsed since the most recent infection, and the presence of cognitive symptoms such as confusion, slowed thinking, word-finding difficulties, and delayed reactions.

Participants who reported experiencing brain fog after their most recent COVID-19 infection rated its perceived severity and impact on daily functioning using a visual analogue scale (VAS) ranging from 0 (no impact) to 10 (maximum impact). The duration of brain fog symptoms was reported in weeks.

The full, unfilled questionnaire is provided as Supplementary File S1.

### 2.4. Human Participants (Ethics and Consent)

This study was approved by the Ethics Committee of the Health Centre of Primorje-Gorski Kotar County (Croatia), the Ethics Committee of the Health Centre of Banja Luka (Bosnia and Herzegovina), and the Ethics Committee of the Health Centre of Novi Sad (Serbia). All study procedures were conducted in accordance with the ethical principles outlined in the Declaration of Helsinki and relevant national regulations.

Participants were fully informed about the purpose, procedures, and voluntary nature of the study prior to participation. Verbal informed consent was obtained from all participants before inclusion in the study.

### 2.5. Data Analysis

Statistical analyses were performed using GraphPad Prism version 8.0.0 (GraphPad Software, San Diego, CA, USA). Data distribution normality was assessed using the Kolmogorov–Smirnov test. Depending on data distribution and comparison type, statistical analyses included one-way analysis of variance (ANOVA), Kruskal–Wallis tests, Mann–Whitney U tests, and chi-square tests with Yates’ correction.

Reaction time outcomes were analyzed in relation to age, gender, COVID-19 status, presence of brain fog, and time elapsed since COVID-19 infection. Statistical significance was defined as a *p*-value < 0.05.

## 3. Results

### 3.1. Sample Description

In total, 613 participants without any prior cognitive impairments or conditions potentially affecting RT were involved in this study. They were categorized into age groups as follows: N (18–30 years) = 150, N (31–40 years) = 159, N (41–50 years) = 149, and N (51–60 years) = 141. After assessing their performance on the Mini-Cog test, 14 participants were found ineligible to continue. The distribution of participants across age and gender groups was uniform (age group distribution: ANOVA, *p* = 0.386; gender distribution: ANOVA, *p* = 0.9996), as illustrated in Table 1.

### 3.2. Data Quality Assessment

**Figure 1:**
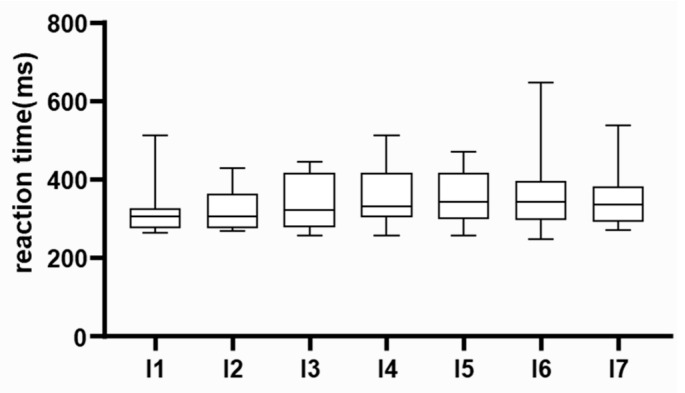
Data quality assessment. Preliminary results were compared among researchers. Median, interquartile range, and absolute range of results are displayed. Individual researchers are labelled on the *x*-axis.

Considering that the study encompassed multiple family physicians practicing in remote clinics, utilizing varied computer systems, and potentially experiencing discrepancies in internet access capabilities, our initial objective was to scrutinize potential variations in the outcomes obtained by different researchers. However, no differences were observed in the results between individual researchers (Kruskal–Wallis test, *p* = 0.1101). These findings are depicted in Figure 1

### 3.3. Prevalence of Brain Fog After COVID-19

**Figure 2:**
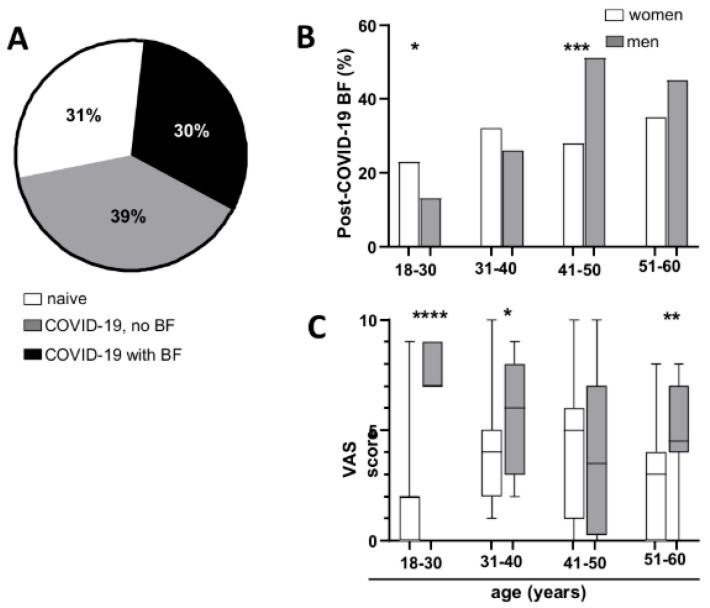
Prevalence of cognitive dysfunction following COVID-19 illness. (**A**) Percentage of subjects who had COVID-19 and reported the presence of BF and (**B**) distribution of post-COVID-19 BF across age and gender groups. (**C**) Bothersome level on a VAS. Median, interquartile range, and absolute range are shown. Statistical significance is indicated by asterisks (* *p* < 0.05, ** *p* < 0.01, *** *p* < 0.001, **** *p* < 0.0001).

We examined the occurrence of BF among individuals who had recovered from COVID-19 and self-reported experiencing BF following their most recent SARS-CoV-2 infection. The findings were compared across different age and gender categories. In total, 44% (N = 186) of those who had COVID-19 reported experiencing BF after their latest infection (30% of all participants) (Figure 2A). The self-reported presence of BF did not show a significant difference by gender among participants. (men 47.7%, women 42%, χ^2^ with Yates’ correction (DF = 1, N = 420) = 0.8979, *p* = 0.3434). Men above 40 years reported the presence of BF at a higher percentage than women (Figure 2B) (Chi-square test, for age groups, respectively: 18–30 χ^2^(DF = 1, N = 150) = 5.064, *p* = 0.0244; 31–40 χ^2^(DF = 1, N = 159) = 1.386, *p* = 0.239; 41–50 χ^2^(DF = 1, N = 149) = 16.436, *p* = 0.0001; 51–60 χ^2^(DF = 1, N = 141) = 2.927, *p* = 0.0871).

### 3.4. Bothersome

Each participant who reported experiencing BF following their most recent COVID-19 infection was requested to assess the degree of bother caused by BF using a visual analogue scale (VAS) ranging from 1 to 10. As illustrated in Figure 2C, men reported higher levels of bother compared to women (Mann–Whitney test, for age groups, respectively: 18–30 *p* < 0.0001, 31–40 *p* = 0.0373, 41–50 *p* = 0.6947, 51–60 *p* = 0.0096).

### 3.5. Differences in Simple and Complex Reaction Time Relative to Age and Gender

**Figure 3:**
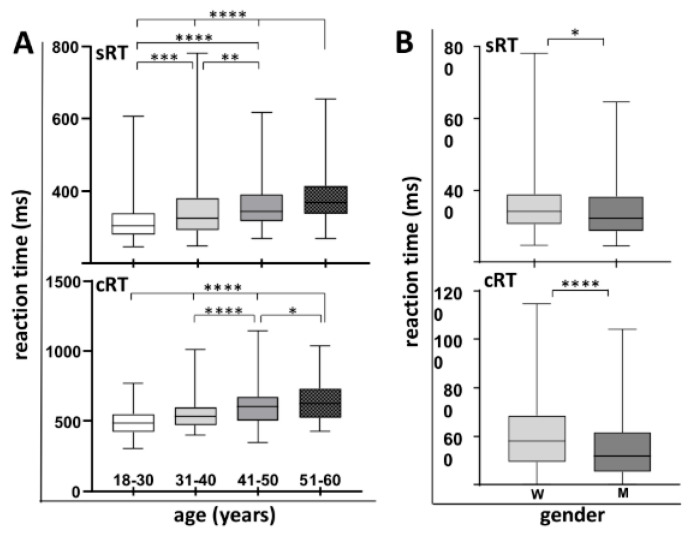
Reaction time relative to age and gender. (**A**) sRT and cRT depending on age. (**B**) sRT and cRT depending on gender. Median, interquartile range, and absolute range are displayed. Statistical significance is indicated by asterisks (* *p* < 0.05, ** *p* < 0.01, *** *p* < 0.001, **** *p* < 0.0001).

We investigated whether there were variations in sRT and cRT across participants, considering their age and gender. As depicted in Figure 3, the results revealed a significant increase in both sRT and cRT across successive age groups (Kruskal–Wallis test, *p* < 0.0001, with post hoc analysis using the Mann–Whitney test presented in Table 2). Additionally, men exhibited shorter RT in both tests compared to women within the same age group (Mann–Whitney test, *p* = 0.0031 for sRT, *p* < 0.0001 for cRT). However, given the uniform distribution of participants by age and gender, as well as the consistent prevalence of COVID-19 infection across different demographics (), the sample can be considered statistically homogeneous.

### 3.6. Reaction Times in Relation to COVID-19 Recovery and Reported Brain Fog

**Figure 4:**
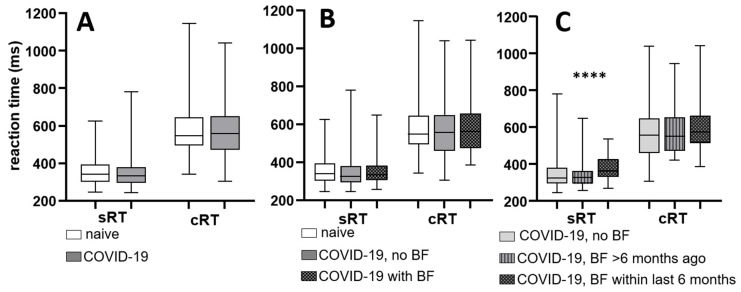
Reaction time relative to COVID-19 infection and the presence of BF. Median, interquartile range, and absolute range are displayed for sRT and cRT relative to (**A**) COVID-19 infection, (**B**) report of BF, and (**C**) time elapsed after BF. Statistical significance is indicated by asterisks (**** *p* < 0.0001).

Participants underwent RT tests involving simple and complex stimuli, and the data were analyzed to investigate any association between COVID-19 recovery and changes in RT. Despite expectations of potential cognitive effects following virus recovery, the findings revealed no statistically significant difference in RT between individuals who had experienced COVID-19 and those who had not (Mann–Whitney test, for sRT *p* = 0.19; for cRT *p* = 0.615) (Figure 4A).

A comparison was made between RT to simple and complex stimuli among individuals who reported experiencing BF after their most recent COVID-19 infection and those who did not. The control group comprised individuals who had never had COVID-19. The analysis revealed no significant difference in RT between the groups (Kruskal–Wallis test, for simple RT *p* = 0.3293; for complex RT *p* = 0.3896). These results are depicted in Figure 4. However, when examining RT based on the timing of individuals’ experience of BF, it was found that RT was significantly impaired in those who had experienced BF within the last 6 months. (Kruskal–Wallis test, for sRT *p* < 0.0001, for cRT *p* = 0.3173).

### 3.7. Changes in Reaction Time over Time Following COVID-19 Infection

**Figure 5:**
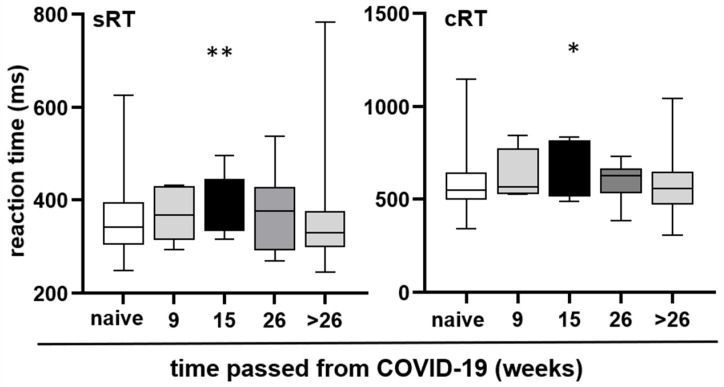
Reaction time at different time intervals after COVID-19. Median, interquartile range, and absolute range are displayed for sRT and cRT relative to the time elapsed after the last COVID-19 illness. Statistical significance is indicated by asterisks (* *p* < 0.05, ** *p* < 0.01).

Because the reporting of BF is subjective and we aimed to provide objective measures, when we found that RT deteriorated in individuals with COVID-19 related BF, we chose to focus our analysis on RT based on the time passed since their last COVID-19 infection, with intervals ranging up to 9 weeks, 15 weeks, 26 weeks (6 months), and beyond 26 weeks. Regardless of whether participants experienced BF or not, both sRT and cRT increased after COVID-19 illness, peaking at 15 weeks post-recovery. Subsequently, it decreased, eventually reaching a similar level as the reaction time of the control group (individuals who did not contract COVID-19) after 26 weeks (Kruskal–Wallis test, for sRT *p* = 0.0054, for cRT *p* = 0.0289). The results are depicted in Figure 5.

### 3.8. Correlation Between Age, Brain Fog Severity, and Reaction Time

To further elucidate the relationship between age, sex, objective cognitive performance, and subjective post-COVID brain fog (BF), correlation analyses were performed in the full sample and separately for females and males. Simple reaction time (simple RT), choice reaction time (choice RT), presence of brain fog after the last infection (Q7), and the degree to which brain fog interfered with work (Q8) and daily life (Q9) were analyzed.

#### 3.8.1. Age and Reaction Times

In the total sample, age was moderately to strongly positively correlated with reaction times, indicating slower responses with increasing age. Significant correlations were observed for both simple RT (Pearson r ≈ 0.51, *p* < 0.01) and choice RT (Pearson r ≈ 0.49, *p* < 0.01), with consistent results obtained using Spearman’s rank correlations.

**Figure 6:**
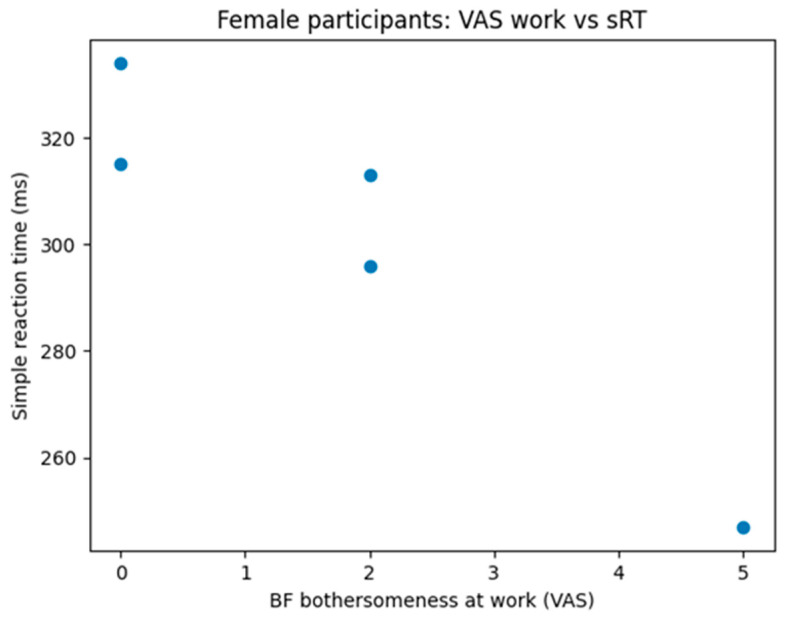
Correlation between perceived brain fog bothersomeness and simple reaction time in female participants. Scatter plot shows individual data points. Spearman correlation analysis revealed a strong negative association between brain fog interference and simple reaction time (ρ = −0.95, *p* = 0.014).

Sex-stratified analyses revealed differences in the strength of these associations. In males, age showed strong and statistically significant correlations with both simple RT (r ≈ 0.68, *p* < 0.05) and choice RT (r ≈ 0.72, *p* < 0.01). In females, the direction of the associations was comparable, with older age associated with slower reaction times; however, these correlations did not reach statistical significance and showed only trend-level effects, likely reflecting limited statistical power (Figure 6).

#### 3.8.2. Age and Presence of Brain Fog

In females, a negative association between age and the presence of brain fog after the last COVID-19 infection (Q7) was observed, indicating that younger women more frequently reported brain fog. This association approached statistical significance (r ≈ −0.50, *p* ≈ 0.06). In contrast, males exhibited a positive and statistically significant association, with older men more likely to report brain fog (r ≈ 0.76, *p* < 0.01).

#### 3.8.3. Age and “Bothersome” Brain Fog Parameters

Across the full sample, no significant correlations were found between age and the degree to which brain fog interfered with work (Q8) or daily life (Q9). A similar pattern was observed in females, where age was not significantly related to either bothersome parameter.

In males, however, age was strongly and positively correlated with work-related interference due to brain fog (Q8) (r ≈ 0.85, *p* < 0.05), suggesting that older men experience a greater functional impact of brain fog in occupational settings. The association between age and interference with daily life (Q9) in males was positive but did not reach statistical significance.

#### 3.8.4. Subjective Brain Fog Severity and Objective Cognitive Performance (Exploratory)

Exploratory analyses examining the relationship between subjective brain fog severity (Q8, Q9) and objective reaction time measures indicated that subjective functional impairment does not uniformly map onto objective cognitive slowing. In females, greater work-related interference was associated with faster simple reaction times, whereas in males, greater interference at work was associated with slower choice reaction times. Given the small number of participants with complete data for these variables, these findings should be interpreted as preliminary.

## 4. Discussion

The present study examined reaction time (RT) and self-reported brain fog (BF) in a large, non-hospitalized adult population following SARS-CoV-2 infection, with the aim of clarifying the temporal dynamics of cognitive impairment and its functional relevance. By focusing on individuals aged 18–60 years without pre-existing neurological disease, the study sought to minimize confounding from age-related cognitive decline and chronic comorbidities known to influence RT [14,15,16]. While this approach strengthened internal validity, it also limits generalizability to older populations, who may experience more pronounced or persistent post-COVID cognitive sequelae.

A substantial proportion of participants (44%) reported BF following COVID-19 infection, underscoring its clinical relevance even among non-hospitalized individuals. Reported prevalence rates of BF in the literature vary widely, likely reflecting differences in study design, symptom definitions, follow-up duration, and patient populations. Meta-analytic data suggest BF prevalence rates of approximately 23–27% at 12 months post-infection, with higher rates observed over longer follow-up periods, particularly among non-hospitalized patients [17]. In contrast, studies focusing on hospitalized or post-acute care populations report a much broader range, from below 10% to over 70% [18,19,20,21]. The prevalence observed in the present study aligns with the upper range reported in community-based samples and supports the notion that BF is not restricted to severe or hospitalized COVID-19 cases.

Sex- and age-specific analyses revealed distinct patterns. While overall BF prevalence did not differ significantly between men and women, younger women (<40 years) more frequently reported BF, whereas older men (>40 years) reported it more often. Previous studies have reported inconsistent sex differences, with some suggesting a higher prevalence in women [18,19,20,21,22], while others indicate that sex effects depend on symptom clusters, timing, and methodological factors [23]. The current findings suggest that age may modulate sex-related vulnerability to BF, emphasizing the importance of stratified analyses when studying post-COVID cognitive symptoms.

Beyond prevalence, BF was associated with meaningful functional impact. Men reported higher levels of BF-related bother across most age groups, despite similar prevalence rates. This discrepancy between symptom frequency and perceived burden may reflect differences in occupational demands, coping strategies, or social expectations, particularly in working-age men. These findings reinforce the value of incorporating patient-reported outcome measures alongside objective cognitive testing to capture the multidimensional nature of post-COVID cognitive impairment.

Reaction time performance followed expected demographic patterns. Older participants demonstrated slower RTs, and men responded faster than women, consistent with well-established literature on age- and sex-related differences in cognitive processing speed [24,25,26,27,28]. This concordance with prior findings supports the validity of the RT assessment methodology used in the study. Importantly, these baseline demographic effects were accounted for in subsequent analyses examining COVID-19-related changes.

At the group level, no persistent differences in RT were observed between individuals with and without a history of COVID-19 infection. This finding aligns with some studies reporting normalization of cognitive performance several months post-infection [29,30,31,32], but contrasts with others that have identified prolonged RT slowing, particularly in hospitalized patients or those with elevated inflammatory markers [32,33]. Notably, the present study detected a distinct temporal pattern: RT impairment peaked approximately 15 weeks after infection and gradually resolved by six months post-recovery. This suggests a transient post-viral cognitive effect rather than permanent impairment.

The relationship between subjective BF and objective RT measures was complex. Overall, BF presence did not consistently predict slower RT, highlighting a partial dissociation between perceived cognitive symptoms and objective performance. However, participants who had experienced BF within the previous six months exhibited significantly slower simple RT, suggesting that subjective symptoms may be most closely linked to objective impairment during the subacute recovery phase. The absence of a similar effect for complex RT may reflect greater interindividual variability or limited statistical power in this subgroup.

Correlation analyses further illustrated the nuanced interplay between age, sex, subjective symptoms, and objective cognition. Age was strongly associated with RT slowing in the overall sample and particularly among men, whereas younger women more frequently reported BF despite weaker associations with objective slowing. Exploratory analyses suggested that subjective interference with work or daily life does not uniformly correspond to objective cognitive slowing, reinforcing the idea that BF encompasses psychological, attentional, and functional dimensions beyond measurable processing speed alone.

Several strengths enhance the interpretability of these findings. The large sample size, multicenter design, and inclusion of participants from three urban regions improve representativeness within the working-age population. Strict exclusion criteria reduced confounding from known neurological and psychiatric conditions, and standardized RT testing ensured methodological consistency. Nevertheless, important limitations should be acknowledged. Self-reported BF is susceptible to recall and perception bias, and individuals with more severe post-COVID symptoms may have been underrepresented due to reduced motivation or fatigue. Additionally, exclusion of participants older than 60 years limits applicability to older adults, a group potentially at higher risk for persistent cognitive sequelae.

In summary, this study supports the view that SARS-CoV-2 infection can lead to transient cognitive slowing, detectable through reaction time assessment, even in non-hospitalized individuals. The findings highlight a critical post-infection window during which cognitive effects are most pronounced and underscore the importance of integrating objective cognitive measures with patient-reported outcomes within a biopsychosocial framework of post-COVID care.

## 5. Conclusions

This multicenter study provides robust evidence that SARS-CoV-2 infection is associated with transient but measurable impairment in cognitive processing speed, as reflected by changes in reaction time (RT), even among non-hospitalized adults without pre-existing neurological disease. Reaction time slowing peaked approximately 15 weeks after COVID-19 infection and gradually normalized by six months post-recovery, suggesting a time-limited post-viral cognitive effect rather than permanent impairment.

Importantly, RT impairment occurred independently of self-reported brain fog, indicating that subjective cognitive complaints and objective cognitive performance do not fully overlap. While brain fog was reported by nearly half of individuals recovering from COVID-19 and was associated with substantial disruption of daily functioning—particularly among middle-aged men—its presence alone did not consistently predict objective slowing of reaction time. These findings underscore the multidimensional nature of post-COVID cognitive sequelae, in which psychological, neurological, and functional components may follow partially distinct trajectories.

The observed age- and sex-related differences further highlight the need for individualized clinical assessment. Older age was consistently associated with slower reaction times, while men demonstrated faster baseline RTs but reported greater functional impact of brain fog, particularly in occupational settings. These patterns suggest that demographic factors may modulate both vulnerability to post-COVID cognitive symptoms and their perceived burden.

From a clinical perspective, this study supports the integration of simple, accessible reaction time testing into routine primary care follow-up of patients recovering from COVID-19. RT assessment offers an objective, low-cost tool for detecting subtle cognitive changes that may not be captured through patient self-report alone. Early identification of post-COVID cognitive slowing could facilitate timely counselling, reassurance regarding expected recovery, and targeted monitoring of individuals at higher risk of prolonged impairment.

Finally, these findings reinforce the importance of a biopsychosocial approach to post-COVID care. Effective management of brain fog and related cognitive symptoms should combine objective assessment, patient-reported outcomes, and psychosocial support. Future longitudinal studies—particularly those including older adults and vulnerable populations—are needed to clarify long-term trajectories, underlying mechanisms, and potential interventions to mitigate post-COVID cognitive dysfunction.

## Figures and Tables

**Table 1:** Subjects Distribution by age and gender.

Age	Total	Men	Women
18–30	150	55	95
31–40	159	57	102
41–50	149	53	96
51–60	141	49	92

**Table 2:** Post hoc analysis of differences in reaction times relative to age and gender of subjects.

By Age	18–30	31–40	41–50	51–60
**18–30**	–	0.0027	<0.0001	<0.0001
**31–40**	<0.0001	–	0.0055	<0.0001
**41–50**	<0.0001	<0.0001	–	0.0017
**51–60**	<0.0001	<0.0001	0.0546	–

by gender: for sRT *p* = 0.0014, for cRT *p* < 0.0001.Grey-shaded rows represent cRT cases; unshaded (white) rows represent sRT cases.

## Data Availability

The datasets used and analyzed during the current study are available from the corresponding author upon request.

## Supplementary Materials

The following supporting information can be downloaded at: https://www.mdpi.com/article/10.3390/neurolint18010006/s1, Supplementary File S1. Brain Fog Self-Assessment Questionnaire (unfilled).

## Abbreviations

ACE2	Angiotensin-converting enzyme 2
BF	Brain fog
cRT	Complex reaction time
COVID-19	Coronavirus disease 2019
CRP	C-reactive protein
NIH	National Institutes of Health
PASC	Post-acute sequelae of SARS-CoV-2
RT	Reaction time
sRT	Simple reaction time
SARS-CoV-2	Severe acute respiratory syndrome coronavirus 2
VAS	Visual analogue scale
