# Supplementary material for: Long-Term Health Consequences of SARS-CoV-2: Reaction Time and Brain Fog
Source: Neurol Int. 2025 Dec 26;18(1):6. doi: 10.3390/neurolint18010006 (PMC12845382; doi:10.3390/neurolint18010006)
Supplement: Supplementary file 1 [file neurolint-18-00006-s001.zip › Supplementary_File_S1_BrainFogQuestionnaire.pdf]

Supplementary File S1. Brain Fog Self-Assessment Questionnaire (unfilled)

Instructions: Please answer the questions below. If you have never had COVID-19, answer "0" for the number of infections and skip items that refer to post-COVID symptoms.

1. How many times have you had COVID-19? (0–4 times)

2. Please describe the severity of the clinical symptoms for each COVID-19 infection. (e.g., mild / moderate / severe; optional free text)

3. How much time has passed since your most recent COVID-19 infection? (weeks or months)

4. Following your most recent COVID-19 infection, did you experience any of the following: confusion, slower thinking, word-finding difficulties, or slower reactions? (Yes/No). If yes, please specify.

5. To what extent did brain fog interfere with your daily work? (VAS 0–10; 0 = not at all, 10 = maximal interference)

6. To what extent did brain fog interfere with your everyday life? (VAS 0–10; 0 = not at all, 10 = maximal interference)

7. How long did the brain fog last? (weeks)
